# Diet, microbiota, and the mucus layer: The guardians of our health

**DOI:** 10.3389/fimmu.2022.953196

**Published:** 2022-09-13

**Authors:** Francesco Suriano, Elisabeth E. L. Nyström, Domenico Sergi, Jenny K. Gustafsson

**Affiliations:** ^1^ Department of Medical Biochemistry and Cell Biology, Institute of Biomedicine, University of Gothenburg, Gothenburg, Sweden; ^2^ Unit for Degradomics of the Protease Web, Institute of Biochemistry, Kiel University, Kiel, Germany; ^3^ Department of Translational Medicine, University of Ferrara, Ferrara, Italy; ^4^ Department of Physiology, Institute of Neuroscience and Physiology, University of Gothenburg, Gothenburg, Sweden

**Keywords:** Mediterranean diet, western diet, fibers, microbiota, bacterial metabolites, mucus layer, intestinal immune system, gut health

## Abstract

The intestinal tract is an ecosystem in which the resident microbiota lives in symbiosis with its host. This symbiotic relationship is key to maintaining overall health, with dietary habits of the host representing one of the main external factors shaping the microbiome-host relationship. Diets high in fiber and low in fat and sugars, as opposed to Western and high-fat diets, have been shown to have a beneficial effect on intestinal health by promoting the growth of beneficial bacteria, improve mucus barrier function and immune tolerance, while inhibiting pro-inflammatory responses and their downstream effects. On the contrary, diets low in fiber and high in fat and sugars have been associated with alterations in microbiota composition/functionality and the subsequent development of chronic diseases such as food allergies, inflammatory bowel disease, and metabolic disease. In this review, we provided an updated overview of the current understanding of the connection between diet, microbiota, and health, with a special focus on the role of Western and high-fat diets in shaping intestinal homeostasis by modulating the gut microbiota.

## Introduction

The gastrointestinal (GI) tract is covered by a single layer of epithelial cells that act as a selectively permeable barrier allowing the absorption of dietary nutrients, microbial metabolites, electrolytes, and water from the lumen into the circulation, while maintaining an effective defense barrier against luminal microorganisms (1, 2). The GI tract harbors a complex and dynamic population of microbes encompassing bacteria, archaea, eukarya and viruses, collectively referred to as the gut microbiota, which has co-evolved with its host in a symbiotic relationship (3, 4). Although the gut microbiota comprises trillions of microbes, the relative sterility of blood and host tissue relies on an intact gut barrier, which acts as the gatekeeper of our health (5). Additionally, apart from acting as a selective barrier, the intestinal epithelium orchestrates the communication between intestinal microbes and the mucosal innate and adaptive immune system (1, 6). The microbiota and its metabolites regulate various aspects of gut immunity, and is thus critical for maintaining mucosal homeostasis (7, 8). The intestinal epithelial cells (IECs) protect the underlying tissues from commensal microbes and/or invading pathogenic microorganisms by secretion of a mucus layer which acts as an additional layer of physical defense of the host, and a habitat for bacteria providing binding sites and energy sources (9). The mucus also acts as a diffusion barrier for anti-microbial peptides (AMPs) released by Paneth cells and other epithelial cells, and immunoglobulin A (IgA) derived from mucosal B cells that prevents microbes from reaching the epithelium (10). Diet is one of the primary factors that influences gut microbiota composition, diversity, and functionality, which in turn have a strong impact on mucus properties, mucosal immunity, and thereby intestinal homeostasis (11, 12). The aim of this review is to summarize the current knowledge regarding the interplay between the diet, microbiota, mucus, and the intestinal immune system with a particular focus on the impact of Western and high-fat (HF) diets on the gut microbiota, and how shifts in the composition and functionality of the microbiota can compromise intestinal homeostasis.

## Gut microbial ecosystem and its composition

The primary colonization of the GI tract begins at birth with the acquisition of microbes from the environment, mainly from the maternal vagina and the skin. The number of microorganisms that reside in the human gastrointestinal tract has been estimated to be 10^13^, a number that is similar to the number of human cells (13). However, the genes encoded by the human gut microbiota, known as the microbiome, are 100-fold more abundant than the genes of the human genome (14). 16S rRNA and metagenomics studies have revealed that the majority of gut microbiota sequences belong to the Bacteria, which is the predominant kingdom in the human adult gut microbiota (15–17). The human gut microbiota is mainly composed by two dominant bacterial phyla: gram-positive Firmicutes and gram-negative Bacteroidetes representing 85-90% of the total microbiota, whereas Actinobacteria, Proteobacteria, Fusobacteria, and Verrucomicrobia are minor costituents (15, 18, 19). Microbial density and diversity increase steadily along the GI tract from the proximal to the distal intestine, a process affected by host features and microbial community dynamics (20). The duodenum harbors 10^3^ microbial cells per gram of intestinal content, and increasing densities/diversities are found in the jejunum (10^4^ cells per gram), ileum (10^7^ cells per gram), and colon (10^12^ cells per gram) (21). Gut microbiota composition also differs transversally from the lumen to the mucosa as demonstrated in both mice and human studies (15, 22, 23). In physiological conditions, the microbiota offers many benefits to the host, which include fortifying the intestinal epithelium, harvesting energy from undigested and unabsorbed nutrients, defending against pathogens, and regulating host immunity (24). However, several environmental (e.g., dietary patterns, antibiotics) and intrinsic (e.g., breast feeding, genetic background) factors can impact the gut microbiota composition and its structural, protective and metabolic functions (11). Additionally, other factors such as oxygen gradients, mucus properties, and the host immune system influence the transversal distribution pattern of the microbiota (23, 25, 26). Over the last years, the microbiota has emerged as a key regulator of host metabolism and health (25). There are several mechanisms by which the microbiota can regulate host metabolism and health, many of which can be ascribed to microbial metabolites (27). Among these bacterial metabolites, the most studied are the short-chain fatty acids (SCFAs) that are produced by bacterial fermentation of indigestible nutrients (i.e., dietary fibers and complex carbohydrates). The role of SCFAs in the regulation of metabolic function and intestinal homeostasis will be detailed in the next sections of this review. In addition to bacteria, another intricate kingdom that co-colonizes the human GI tract is composed of a substantial quantity of viruses collectively referred to as the gut virome (28). Viruses of bacteria, called bacteriophages (phages), are significantly more abundant than eukaryotic viruses, and the estimated phage-bacterial ratio in the human gut is believed to be 1:1 (29). Emerging studies have shown that phage-driven alterations of the microbiota composition by direct interactions or potentially *via* the human immune system have been associated with several diseases (e.g., inflammatory bowel disease, cancer, obesity) (28–30). However, little is known about the phage-mucus interactions, and therefore the present review only covers the mucus-bacteria feedback system of the gut. 

## Diet and gut microbiota: Learning from human and murine models

**Figure 1:**
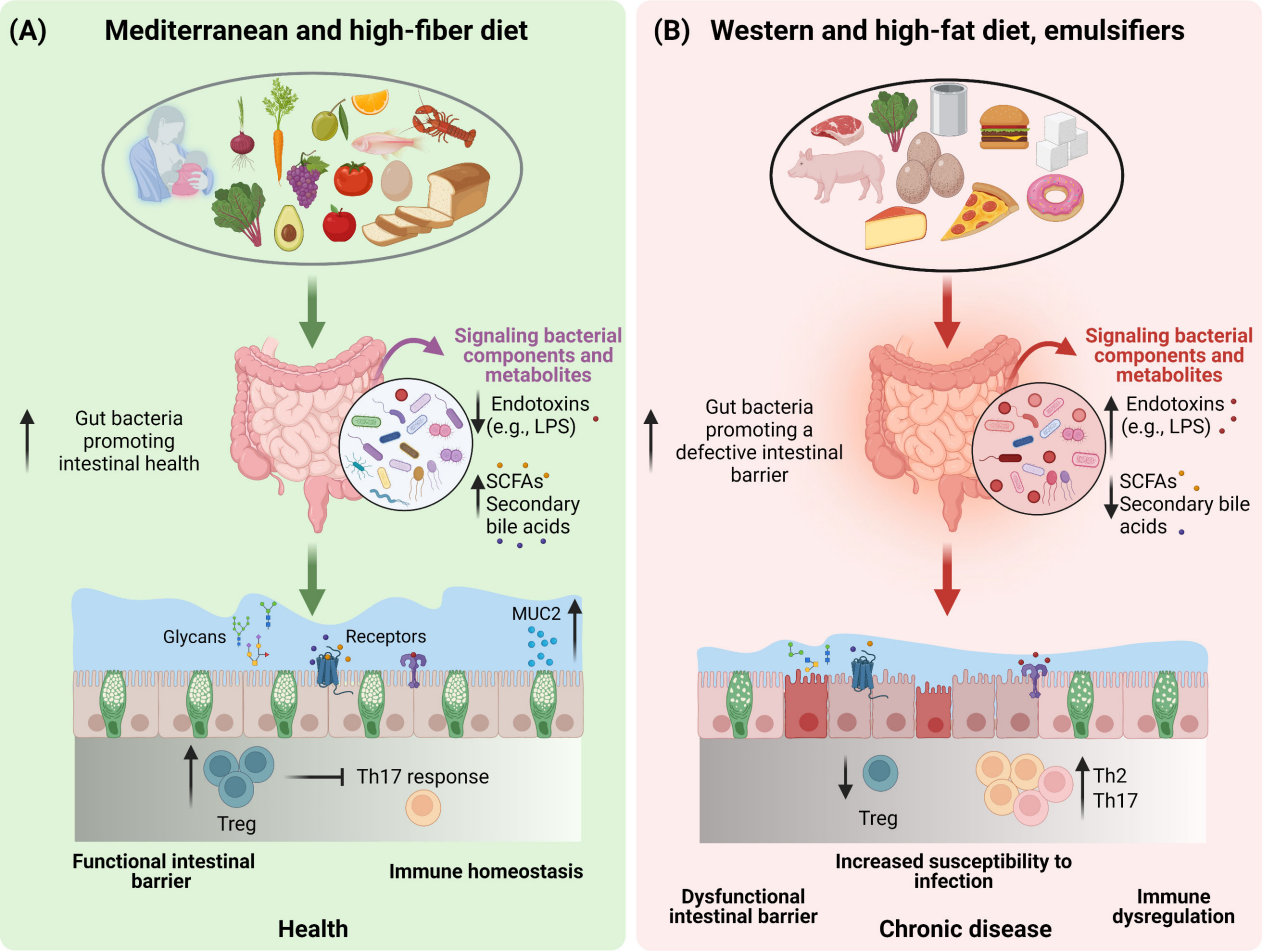
The impact of dietary patterns on gut microbiota and intestinal health. A schematic overview depicting the importance of diet and dietary constituents in discriminating between a healthy **(A)** or an unhealthy **(B)** state of the gut barrier function by modulating the composition and functionality of the gut microbiota.

Diet is a key discriminant in shaping health and aging trajectories with these effects being also mediated by the ability of nutrient quality and quantity to modulate gut microbiota composition and metabolic function (31). Indeed, in addition to providing the energy and the nutrients needed to sustain the cellular processes required for daily life (31), dietary components are also instrumental factors in modulating the microbial communities in the gut (32). In addition to the plethora of the effects exploited by the gut microbiota on host health, the intestinal microbiota community also regulates the mucus barrier function (12, 33). In light of this, dietary-driven modulations of the gut microbiota will also be reflected upon the gut mucus barrier function and the overall intestinal health (34) (**Figures 1A, B** **)**. However, not all diets are equal, and it is established that different dietary patterns exert distinct effects on the gut microbiota. In agreement with this, a diet rich in fiber including galacto- and fructo-oligosaccharides (two important groups of prebiotics) and resistant starch strongly impacts the composition, diversity and metabolic function of the microbiota (35). To this end, dietary fiber provides a plethora of substrates for fermentation reactions carried out by specific species of microbes (e.g., *Bifidobacterium*, *Faecalibacterium*) that express the adequate enzymatic machinery to break down these complex carbohydrates and to produce SCFAs (e.g., acetate, butyrate, propionate). The SCFAs in turn exert beneficial effects on cardio-metabolic (36) and gut health (37), including promoting mucus barrier function (38, 39) (**Figure 1A**). Prompted by these evidences, a move towards a diet high in dietary fiber, low in glycemic index carbohydrates, long-chain saturated fatty acids, animal protein (i.e., red and processed meat), and sugar referred to as the Mediterranean diet (hereafter, MD), has been associated with the prevention of cardiovascular and metabolic diseases, and many other diseases (40, 41). The consumption of a MD has been shown to increase the levels of the fiber-degrading *Faecalibacterium prausnitzii* and genes associated with microbial carbohydrate degradation and butyrate metabolism in a population at risk for cardio-metabolic disease (42). An increased levels of fecal SCFAs, *Prevotella* and fiber degrading *Firmicutes* was also observed in healthy overweight and obese subjects with a high-level adherence to a MD (43). The importance of the dietary fiber (referred to as microbiota accessible carbohydrates, MACs) intake was also demonstrated in mice colonized with a human microbiota and showed that a low-MAC diet resulted in a reduction in microbial diversity in just three generations, which could not be brought back when mice were fed a normal MAC diet (44). Additionally, deprivation of dietary fiber was also associated with deleterious effects on the intestinal mucus layer by the gut microbiota (45–47), further highlighting the importance of dietary fiber for our health and gut microbiota ecosystem.

In contrast to a fiber-rich diet, hypercaloric diets high in long-chain saturated fatty acids and ω-6, sugar and low in dietary fiber, referred to as the Western diet (WD) and HF diet in the case of animal models have not only been widely recognised for their detrimental effects on cardio-metabolic health, but also negatively affect the gut microbiota composition and functionality (11, 48–50) (**Figure 1B**). A key feature of both the WD and the HF-diet, is the high intake of dietary lipids especially in the form of triglycerides. Of note, dietary triglycerides differ in terms of their fatty acid composition (saturated *vs* unsaturated), which in turn represents a further discriminant dictating the effects of lipids on the gut microbiota. In mice, lard, which is rich in long-chain saturated fatty acids, promotes an increase in *Bacteroides* and *Bilophila* as compared to fish oil which is rich in unsaturated fatty acids and particularly ω-3 which promotes an increase in *Bifidobacterium*, *Lactobacillus* and *Akkermansia muciniphila* (51). Despite the majority of the lipids being digested and absorbed in the upper intestine, a high lipid intake, as in the case of HF-diets, in animal models promoted a decrease in bacteria count and a shift in microbiota species abundance (52). Indeed, when given to mice, HF-diets have been reported to increase the Firmicutes to Bacteroidetes ratio (52–54). Additionally, HF-diets have been shown to reduce gut bacteria promoting intestinal health, such as *Akkermansia muciniphila*, *Bifidobacterium* spp., *Bacteroidetes* spp., *Lactobacillus* spp. and *Clostridiales* spp., while increasing gut bacteria associated with defective gut barrier function, such as *Oscillibacter* spp. and *Desulfovibrio* spp (48) (**Figure 1B**). Disrupted gut barrier integrity results in increased gut permeability to luminal bacterial components such as lipopolysaccharide (LPS) resulting in chronic low-grade inflammation typical of obesity and related metabolic comorbidities (55–57). In addition to microbial activation of the intestinal immune system, the inflammation is also fuelled by saturated fatty acids overload, which by themselves are able to elicit pro-inflammatory responses (58). These effects translated in the development of metabolic aberrations, particularly given the role of low-grade chronic inflammation in impairing insulin sensitivity (59). Nevertheless, as already mentioned, WD and HF-diets are generally low in dietary fiber, therefore it is difficult to discern whether the observed effects on the gut microbiota are due to the deprivation of dietary fiber, or the high sugar and fat intake. In response to this conundrum, the effects of a WD and HF-diets on the gut microbiota were mitigated by the supplementation of dietary fibers, further supporting their prominent role in gut health (39, 60–62).

The metabolic activity and composition of the gut microbiota can also be modulated by dietary protein. Diets with a high protein/carbohydrate ratio may exert promising effects in preventing obesity and improving glycemic control as described in animal models (63, 64) and humans (65, 66), even though the effects of these dietary patterns on metabolic health in human remain controversial (67, 68). However, proteins, especially if consumed in excess are able to negatively influence the microbiota. In humans, a high-protein diet was found to decrease butyrate-producing bacteria and fecal butyrate levels (69), and decrease the abundance of beneficial bacteria like *Bifidobacterium*, *Roseburia* and *Eubacterium rectale* (70, 71). Despite these findings, it must be taken into consideration that the impact of protein on the gut microbiota is dictated by the amino acid composition and their relative abundance. Amino acids can in fact be metabolised by the microbiota resulting in the production of a wide array of metabolites which, in turn, affect the health of the host. In line with this, different amino acids exert different effects on the gut microbiota. For example, methionine restriction in mice results in an increase in SCFA-producing bacteria and inflammation-inhibiting bacteria with a concomitant decrease in inflammation-promoting bacteria (72). To the same extent, the protein source represents another variable underpinning the effects of dietary proteins on the gut microbiota. Indeed, vegetable proteins have been shown to increase *Bifidobacterium* and *Lactobacillus*, an effect which may also be dependent on the fact that vegetable source of proteins also represent a source of dietary fiber, as opposed to some animal proteins which combine a lack of fibers with high-levels saturated fatty acids (73).

Besides the role of nutrients, other food ingredients are also emerging as potent modulators of the gut microbiota. Of these, dietary emulsifiers like carboxymethylcellulose and polysorbate-80 have been shown to impact the gut microbiota composition increasing the susceptibility to colitis and the metabolic syndrome in animal models (74). Overall, the changes in the gut microbiota elicited by emulsifiers produced a shift in gut bacteria in a manner to promote and sustain intestinal inflammation (75) (**Figure 1B**). However, although numerous emulsifiers increased the pro-inflammatory potential of the gut microbiota *ex vivo*, these effects were not induced by all commonly used emulsifiers (76). In this regard, carrageenans and gums were shown to alter microbiota density, composition as well as the expression of pro-inflammatory molecules (76). Altogether, findings from both pre-clinical and clinical studies highlight the importance of diet, nutrients, and food ingredients in the modulation of the gut microbiota, resulting in either beneficial or detrimental health outcomes.

## The intestinal barrier

**Figure 2:**
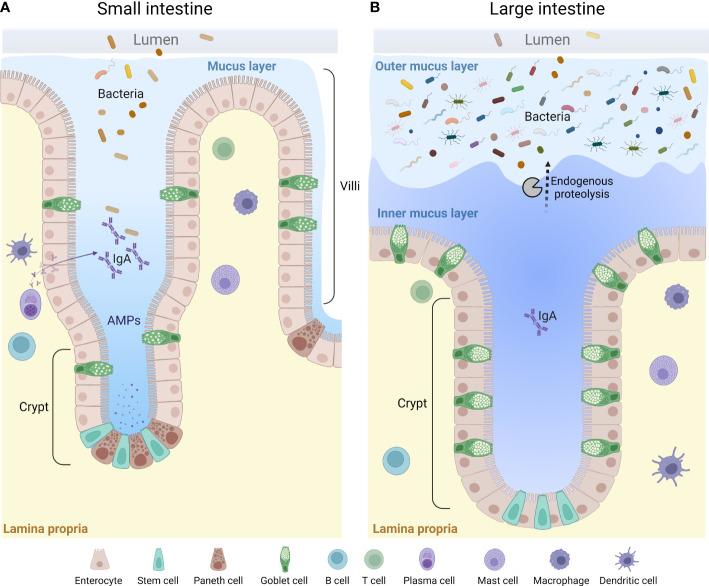
A general overview of the mucus layer in the small and large intestine. The mucus layer is a key component of the intestinal barrier. It is mainly composed of mucin glycoproteins produced and secreted by the intestinal goblet cells. The small intestine is characterized by a loose and unattached mucus layer containing antimicrobial products that limit penetration by bacteria **(A)**, whereas the large intestine presents a dense and firmly attached mucus layer converted to a looser structure at the luminal side *via* endogenous proteolysis **(B)**. AMPs, anti-microbial peptides.

The intestinal barrier is multi-tiered, including the mucus layer, the epithelial layer and the underlying immune system (**Figures 2A, B**). At this interface, appropriate host-microbiome interactions play an important role in maintaining intestinal homeostasis throughout life (77). Disturbance to any of these layers by several factors such as dietary-driven changes in the gut microbiota composition, antibiotics usage, and genetic susceptibility, is associated with the onset of chronic disease including inflammatory bowel disease, extra intestinal autoimmune disease, metabolic disorders such as diabetes and obesity and allergic disease (78, 79). This section of the review will focus on the organization and composition of the different parts of the intestinal barrier in the steady state.

### The mucus layer

The mucus layer, produced by specialised secretory goblet cells (GCs) in the epithelium covers the intestinal tissue to provide lubrication and protection against the luminal material, especially the microbiota. Mucus homeostasis is crucial for health and its dysregulation in either the small intestine or the colon causes or correlates with various diseases. Indeed, mucus accumulation and bacterial overgrowth due to loss of bicarbonate secretion is evident in cystic fibrosis, and lost mucus barrier properties and increased bacterial burden at the epithelium in the colon correlate with the inflammatory bowel disease ulcerative colitis (UC) (80–83). Thus, an understanding of factors that impact mucus homeostasis is needed in order to maintain a good “gut health”. External factors can alter mucus-related properties and as the major external factor affecting the intestine, diet can have profound effects on the mucus barrier. Mucus associated effects induced by the diet can be direct on the mucus or the epithelium (84–87) but perhaps more importantly indirect by influencing the microbiota which in turn can have strong effects on the intestinal mucus, which will be further discussed in the last part of this review (39, 45, 88–91).

The properties and thickness of the mucus layer varies along the GI tract to facilitate the physiological function in each location (33, 92, 93). The small intestinal mucus has been described as loosely organized to allow for efficient nutrient absorption along the full length of the villi protrusions (92, 94) (**Figure 2A**). To maintain a protective barrier against luminal bacteria and digestive enzymes, the small intestinal mucus is fortified by AMPs secreted from crypt-base residing Paneth cells and enterocytes at the base of the villi, IgA secreted by plasma B cells, and endogenous enzyme inhibitors (94–96) (**Figure 2A**). In the small intestine, the thickness of mucus layer covering the follicle associated epithelium of Peyer’s patches has been shown to be thinner as compared to the surrounding epithelium, which likely facilitates antigen and particle uptake by small intestinal M cells (97). Only a thin separating mucus layer covers the proximal colon of mice, and bacteria can be seen in close contact with the epithelium, as evident by histology (92, 98, 99). However, the mechanisms allowing the close proximity of bacteria to the epithelium in the proximal colon remains to be defined (100). As the luminal material solidifies during its distal transport, a separating mucus barrier is formed, distancing the fecal matter and the microbiota from the underlying tissue (98, 99). Thus, in the distal parts of the colon of both human and rodents, the epithelium is protected by a mucus barrier which physically separates the luminal microbiota from the tissue surface (93, 101, 102). However, mucus material can also be seen intermixed with the microbiota in the outer regions of fecal pellets, where it is thought to provide a nutrient rich ecological niche for some bacteria (20, 22, 101). This “inner” and “outer” mucus thus creates a two-layered mucus structure in the distal colon (80, 92, 93, 101) (**Figure 2B**). The conversion from the inner to the outer mucus layer is dependent on endogenous proteolytic activity, but bacterial proteolysis could play an additional role (101, 103) (**Figure 2B**).

### Mucus composition

The mucus is composed by a core set of about 30 proteins, most of which are produced by GCs, including mucin 2 (MUC2), chloride channel accessory-1 (CLCA1), Fc fragment of IgG binding protein (FCGBP) and zymogen granule protein 16 (ZG16) (83), as well as lipids, ions, and water (which makes up for approximately 95%) (104). The MUC2 glycoprotein is the main structural component and creates a net-like scaffolding backbone of the mucus along the GI-tract by oligomerization of its terminal ends (105, 106). MUC2 is a very large protein (>5000 amino acids) with two mucin characteristic PTS-domains, rich in proline, serine and threonine, which becomes heavily *O-*glycosylated by glycosyltransferases in the secretory pathway. The added glycans makes up for approximately 80% of the molecular weight in the mature protein (107). More than 100 different glycan structures have been identified in intestinal MUC2 (108). The distribution of different glycans is region and species specific, adding additional layers of complexity to the mucus barrier (109, 110). Apart from protecting the MUC2 protein backbone from proteolysis and giving MUC2 its gel properties by binding water, the glycans attached to MUC2 provide microbial adhesion sites and a nutritional source in the outer mucus layer (111–114). The glycan composition on MUC2 can thus provide a strategy for the host to select commensal bacteria, but can also be utilized by both commensal and pathogenic bacteria (115–117). Microbiota dependent degradation of mucin glycans induced by a low-fiber diet increases the susceptibility to *Citrobacter rodentium* infection in mice (45), indicating the importance of Muc2 glycosylation in protection against pathogenic bacteria. Shortened and less complex glycans have been identified in patients with active ulcerative colitis (118). It does, however, remain unknown whether the observed alterations are a cause, or an effect of the inflammation, and further investigations are needed to elucidate these mechanisms.

### Mucosa and mucus-associated microbiota

The bacterial diversity of the mucosa-associated bacteria is on par with that of the luminal microbiota, but the composition differs (39, 119). In general, mucosa-associated bacteria have a higher abundance of the phylum Firmicutes compared to Bacteroides both in humans and rodents (120). Although the inner mucus layer appears mostly sterile by histological examination, Bergström et al., detected appreciable levels of bacterial 16S in mucus collected in inter-pellet regions, indicating that some bacteria, especially so called mucus specialists, indeed can colonize this barrier, which was also evident by *ex vivo* imaging (119). Additionally, bacterial analyses of mucosal samples from laxative treated patients indicate their presence in mucus from the different sampling sites in the large intestine, as well as in the distal ileum (121). A couple of studies have demonstrated the presence of a crypt-specific microbiota in both mouse and human colon characterized by a low density of bacterial community dominated by *Acinetobacter* spp. in mouse and generally enriched for Proteobacteria capable of aerobic metabolism in both human and mouse (122, 123). It should however be noted that this was restricted to the proximal colon in mouse, and only affected a small number of colonic crypts in human. It is possible that the number of bacteria in the inner mucus layer and colonic crypts differ between strains/vivariums, reflecting differences in mucus quality controlled by the gut microbiota. Furthermore, in physiological conditions, mucin-degrading specialists (e.g., *Akkermansia muciniphila* and, *Bacteroides thetaiotaomicron*) live in mutual coexistence with host, and the rate of mucus degradation is balanced with the rate of mucus synthesis, resulting in a dynamic and stable mucus structure that is of fundamental importance for our “gut health”. Only few bacterial species have the enzymatic machinery for initiating partial or full mucin degradation, including *A. muciniphila*, *Bacteroides thetaiotaomicron*, *B. bifidum*, *Bacteroides fragilis*, *Ruminococcus gnavus*, and *Ruminococcus torques* (120). These mucin-specialist degrade the mucin protein, which possibly leads to the availability of oligosaccharides for other bacteria that do not harbour the correct enzymes for this process allowing bacterial cross-feeding and mucosal health (47, 120). An example is the interaction between the *B*. *thetaiotaomicron* (*B.theta*) and the *Faecalibacterium prausnitzii*. Wrzosek et al., have shown that *B. theta*, an acetate producer, increased goblet cell differentiation, expression of mucus-related genes, and mucus glycosylation in mono-colonized rats. In contrast when *B.theta*-mono-colonized rats were supplemented with *Faecalibacterium prausnitzii*, an acetate consumer and butyrate producer, the increase in goblet cell differentiation and the alteration in mucus glycan profile was reduced, thus maintaining an appropriate structure and composition on the gut epithelium (124). Moreover, additional studies are needed to elucidate the role of the mucosa-associated microbiota in regulation of intestinal homeostasis as this microbial community is located closely to the host.

### The intestinal immune system

Although the intestinal epithelium with its mucus layer act as the first layers of defense against the potentially harmful agents that pass through our GI tract, this is not an absolute barrier. Indeed, dietary and microbial antigens, and metabolites readily pass the mucus layer and epithelium and enter the lamina propria (LP) where they are sampled and sensed by the intestinal immune system (125, 126). In the intestine, the immunological challenge lies in how to accurately discriminate between harmless and harmful foreign antigens, and failure to respond adequately to the large variety of antigens that pass through the GI tract results in chronic inflammatory conditions such as food allergy and inflammatory bowel diseases (127, 128). The ability of the immune system to mount appropriate responses to the luminal content is to a large extent regulated by the composition of the content itself which influences the immune status of the host. The intestinal immune system can be divided into innate and adaptive immunity, where dendritic cells (DCs), macrophages, neutrophils, mast cells, eosinophils, basophils, natural killer T cells (NKT) and innate lymphoid cells (ILCs) make up the innate arm of immunity, and T and B cells make up the adaptive arm. During steady state, adaptive and innate immune responses in both the small intestine and colon promote tolerance and inhibit pro-inflammatory responses allowing us to live in symbiosis with our microbiota and tolerate the food we eat. Tolerance to the luminal content is to a large extent dependent on induction and maintenance of T regulatory cells (Tregs), a subset of CD4+ T helper cells (Th) that suppresses effector T cell responses, and by plasma cells that secrete large quantities of IgA directed towards luminal antigens (129). Innate immune cells such as macrophages and DCs contribute to maintaining a local environment promoting tolerance and induce adaptive immune responses. Tissue resident LP macrophages produce the anti-inflammatory interleukin (IL)-10, and prostaglandins that promote local tolerance in the LP, and migratory LP-DCs traffic to small intestine and colon draining lymph nodes following antigen acquisition where they promote *de novo* Treg induction (130, 131). Other innate cells such as eosinophils, more known for their role in pathological conditions such and allergic disease (132), were recently shown to contribute to intestinal homeostasis by stimulating and maintaining IgA producing plasma cells, and by regulating LP Treg and DC populations as mice lacking eosinophils were shown to have reduced number of LP Tregs and CD103^+^ tolerogenic DCs (133).

## Microbial regulation of the intestinal homeostasis, lessons from the use of germ-free mice

The role of the microbiota in regulation of the mucus layer is an area of continuous research, and a large proportion of the current knowledge comes from studies using germ free (GF) mice, and mono-colonization or conventionalization of GF mice. In this section we will describe how the absence or the re-introduction of microbes in murine models affect development and regulation of the mucus barrier, and intestinal immunity.

### Mucus and microbes

The first evidence on the importance of microbes in the regulation of the intestinal mucus layers was demonstrated in a conventionalization experiment using GF mice. The authors discovered that in the GF ileum, the mucus was attached to the epithelial surface in contrast to conventionally raised (Convr) mice in which the mucus is easily aspirated, a process shown to be regulated by microbial activation of meprin β, an enzyme involved in detachment and release of mucus in the small intestine (90, 134). In the GF colon, the mucus was thinner and more penetrable to bacterium sized fluorescent beads as compared to Convr mice, and the amount of Muc2 was lower in the GF colon as compared to Convr colon (90). Furthermore, the glycosylation pattern of Muc2, the overall expression levels of glycosyltransferases, and the length of the glycans were shown to differ between the two groups (90, 110). It has also been shown that single bacterial species (i.e., *B. theta* and *Faecalibacterium prausnitzii*) can promote colonic epithelial homeostasis by modifying goblet cell differentiation, expression of mucus-related genes, and mucin glycosylation (124). Johansson at al., have also shown that gut microbiota colonization of GF mice, and normalization of mucus properties is a slow process (90). Their results showed that it takes about 6 weeks for the colon inner mucus layer to become fully impenetrable to bacteria and bacteria sized beads, and 8 weeks for the microbiota to reach the composition of Convr mice (90). Noticeably, a normalizing change in several mucus parameters correlated with a shift in the ratio between Firmicutes and Bacteroides in the mucus (90). The importance of the microbiota in modulating the mucus phenotype was also illustrated in a comparison of two C57BL/6 mouse colonies with the same genetic background housed in different rooms of the same vivarium. The two colonies were characterized by a divergence in their gut microbiota composition as well as in their mucus phenotypes (88). One colony had mucus that was impenetrable to bacteria or bacterium sized fluorescent beads, whereas the other colony had an inner mucus layer penetrable to bacteria and beads (88). These divergences were traced back to the gut microbiota, since transfer of cecal microbiota from the two colonies to GF mice, was able to transfer the respective mucus properties. Analysis of the gut microbiota composition demonstrated that mice with an impenetrable mucus layer had increased amounts of the Erysipelotrichi class (mainly the genus *Allobaculum*), whereas mice with a penetrable mucus layer had increased levels of Proteobacteria and TM7 bacteria in the distal colon mucus (88). In a study of inflammasome-deficient mice, the mucus barrier function in *Il18-/-* mice was found to be dependent of the microbiota and could be either transferred or lost upon co-housing with different wild-type mice. Two fecal bacteria strains: the Bacteroidales family S24-7 (Muribaculaceae) and the genus *Adlercrutzia* were identified to be consistently and positively correlated with inner mucus layer function (89). Volk et al., have also provided further detailed information regarding correlative and causative relations between bacteria and mucus properties when pooling public dataset of different experiments (89). Additionally, feeding mice a WD induced a bloom of the Proteobacteria *Helicobacter* and a lower relative abundance of S24-7 family and Bifidobacteria, which correlated with a microbiota-dependent loss of mucus barrier function (39). Re-introduction of Bifidobacteria corrected certain aspects of the mucus dysfunction, but did not completely restore the mucus properties. These findings through a pre-clinical approach highlight three important points: (i) it is the selective increase in certain bacterial species and their specific functions rather than changes to the entire microbial community that regulates the properties of the inner colon mucus barrier, (ii) housing conditions are critical cofounding factors when investigating microbe-mucus interactions, and a standardized approach should be considered when comparing animal studies, (iii) time is of importance when analyzing bacterial-host interaction in GF and Convr mice.

The mechanisms by which microorganisms regulate mucus properties involve both bacterial metabolites such as SCFAs and secondary bile acids, and microbial components that bind pattern recognition receptors such as toll-like receptors (TLRs) expressed by GCs (135). Among the several SCFAs, butyrate has been shown to increase the production of MUC2 in cultured intestinal epithelial cells (136), and stimulates mucus release from the rat colon (86, 137). In addition to SCFAs, other classes of bacterial metabolites involved in the regulation of the mucus properties are the secondary bile acids. One example is deoxycholic acid (DCA), which has been shown to induce MUC2 expression in cultured colonic epithelial cells (138, 139). However, there are many controversial results associating bile acids with improvement of gut barrier function, and further investigations are required to elucidate their role in regulation of gut health (2). Like the bacteria metabolites, exposure of colonic tissues to high concentrations of bacterial products such as LPS and peptidoglycan (PGN), induces mucus secretion in both GF and Convr mice (140). Furthermore, mice lacking the TLR adaptor protein MyD88 present a decreased production of mucus, impaired goblet cell responses and reduced antimicrobial activity against *Citrobacter rodentium* infection (141). To the same extent, mice lacking the flagellin receptor, TLR5 deficient mice, have a disorganized mucus structure as compared to wild-type mice and an increased abundance of Proteobacteria in close contact with the epithelial surface (26). Altogether, these *in vitro* and *in vivo* studies indicate the importance of microbes or their metabolites and components, and host TLRs in maintaining mucosal health.

### Microbial regulation of intestinal immunity

It is well established that adaptive immune responses are immature in both the small and large intestine of GF as compared to Convr mice. GF mice have reduced size of Peyer’s patches and reduced numbers of IgA producing plasma cells, LP T cells, and intra epithelial lymphocytes (IELs) (142–144). With respect to innate immunity, less is known about the role of the microbiota in regulation of the respective cell types, and observations differ between studies, possibly related to variations in the microbiota composition of the Convr control group. Colonic macrophages, DCs, and mast cells have been reported to be immature and reduced in numbers in GF mice as compared to Convr mice, suggesting a stimulatory role of the microbiota in driving proliferation and maturation of these cell types (145, 146). In contrast, eosinophil and NKT cell numbers have been reported to be increased in GF mice pointing towards a suppressive role of the microbiota in regulation of these cells (147). However, despite the observed increase in eosinophil numbers in GF mice, the cells appear inactive as they express less of eosinophil peroxidase (148, 149).

To further dissect the role of specific members of the microbiota in regulation of gut immunity, mono-colonization or colonization of GF mice with a limited consortium of bacteria have been used to study the role of the microbiota in regulation of both adaptive and innate immunity. In the context of microbial regulation of tolerance to the luminal content, studies have demonstrated that bacteria from the *Clostridium* genus cluster IV and XIVa promote induction of FoxP3^+^ Tregs in the colon (150). As mentioned previously, *Clostridia* species are well known for their ability to metabolize dietary fibers into SCFAs, and catabolize tryptophan into the ahryl carbon receptor (AhR) ligands indole and indole derivates, and it is considered that most effects of *Clostridia* on regulation of intestinal immunity is mediated *via* these metabolites. SCFAs regulate immune cell function by two main pathways 1) by activating G protein coupled receptors (GPRs): GPR41, 43 and 109A, and by acting as histone deacetylase (HDAC) inhibitors. HDACs are enzymes that regulate gene expression and thereby affect a variety of functions including proliferation and differentiation (151, 152). In the colon, SCFAs promote induction of Foxp3^+^ Tregs *via* HDAC inhibition, and by activation of GPR43 (153, 154). Feeding GF mice with SCFAs was shown to increase FoxP3 and Il10 expression, and increase the suppressive effect of Tregs (155). Butyrate has also been shown to inhibit the Th17 transcription factor RoRγt, and IL-17 production *in vitro* (156). Thus, in the colon, SCFAs promote tolerance *via* Treg induction and inhibition of Th17 responses. In contrast, SCFAs have not been shown to induce Treg responses in the small intestine, however other bacterial metabolites such as secondary bile acids (e.g., 3-oxolithocholic acid) and tryptophan catabolites (e.g., indole) that bind the AhR stimulate Treg induction and inhibit Th17 responses in both the small intestine and colon (157–159). SCFAs have also been shown to stimulate IgA production by intestinal B cells, and increase LP-DC expression of indoleamine 2,3-dioxygenase 1 (IDO1) and aldehyde dehydrogenase 1A2 (Aldh1A2) which promote conversion of naïve T cells into Tregs, thus, further promoting an environment favoring tolerance over inflammation (160, 161). Collectively these findings underscored the importance of the microbiota in regulation of maturation of intestinal immunity and immune homeostasis.

## Dietary patterns: The determining factor for the intestinal homeostasis

It is now well established that early nutrition can influence the development of the gut microbiota (162), and immediately after birth, breast milk or infant formula or a combination of both is our primary diet. Human milk oligosaccharides (HMOs) which are the most abundant components in breast milk cannot be digested by the human infant, but because of their structural similarity to mucin-glycans, they can be used as a primary carbon source by several bacterial strains (e.g., *Bifidobacterium* species, members from the genus *Bacteroides*) implicated in the initial colonization of our intestine, with beneficial effect on our mucosal, immune, and metabolic health during later life (163, 164). Both *in vitro* and *in vivo* studies have highlighted the ability of HMOs and HMO-compounds (i.e., 2′ -fucosyllactose) to modulate mucins expression and the secretory function of GCs (165, 166). HMOs can directly control intestinal immunity by decoying receptors of pathogenic bacteria and viruses, thereby preventing their binding on intestinal cells and the onset of a disease (167). Transitioning from milk based to solid food, and the introduction of fiber to our diet, the pivotal metabolic substrate for the gut microbiota, induce the production of SCFAs. As mentioned previously, these gut-microbiota-derived metabolites and especially butyrate can improve mucus barrier function *via* modulation of MUC2 production and expression (26) (**Figure 1A**). Moreover these bacterial metabolites act as ligands for GPR43, GPR41, and GPR109A that in addition to being expressed by immune cells also are expressed by epithelial cell, primarily cells of the secretory lineage (168). Activation of these receptors triggers the release of enteric peptide hormones including glucagon-like peptides 1 and 2 and peptide YY, which regulate several metabolic functions such as improvement of the gut barrier, metabolic inflammation, and gut transit time (25).

In contrast to diets high in dietary fiber that promote intestinal health, WD and HF-diets that often are low in dietary fiber, have been associated with loss of mucus barrier function, impaired immune homeostasis, and increased susceptibility to chronic inflammatory diseases including inflammatory bowel diseases and food allergies (46, 169). These effects have largely been related to loss of SCFAs production, but studies have also demonstrated a direct toxic effect of dietary fatty acids on T cells *in vitro* (170). As mentioned previously, high intake of WD and HF diet, is associated with an altered and less diverse gut microbiota composition (48, 171), which in turn contribute to an impaired mucus barrier (39, 172). Similar observations have been made in mice treated with dietary emulsifiers (74, 173) (**Figure 1B**). Lack of dietary fiber induces a shift in the gut microbiota composition toward the utilization of host-glycans such as those provided by mucins as energy source, with deleterious consequences on the mucus barrier (174, 175), and with an expansion and activity of colonic mucus degrading bacteria (i.e., *Akkermansia muciniphila* and *Bacteroides caccae*) and a decrease in fiber-degrading bacteria (i.e., *Eubacterium rectale* and *Bacteroides ovatus*) both in fecal samples, the colonic lumen, and the mucus layer, resulting in an increased susceptibility to gastrointestinal pathogen infections in mice (45) (**Figure 1B**). Nowadays, particular attention is given to the mucin specialist *Akkermansia muciniphila*, whose abundance is reduced in mice exposed to increased dietary fat content. This observation is of interest, since *Akkermansia muciniphila* supplementation in both mice and humans has been linked to improved health outcomes and gut barrier function (176, 177), further reinforcing the idea that the presence of certain bacteria in our gut is of fundamental importance for our health. With respect to the effect of WD and HF diets on intestinal immunity, HF diet has been associated with loss of both Treg and Th17 populations in the small intestine, while in the colon HF diet has been associated with Th2 skewing, and increased susceptibility to allergic disease (178–180) (**Figure 1B**). Altogether, these findings emphasize that dietary patterns, bacteria and bacterial components contribute to maintaining gut homeostasis.

## Conclusions and future perspectives

In this review we have highlighted the importance of the interplay between the resident microbiota and the host in regulation of intestinal homeostasis, and how this interplay is influenced by the dietary habits of the host. It is well established that diets with a high fiber content, and low amounts of fat and sugar promote a microbiota that has beneficial effect on intestinal health by stimulating intestinal mucus barrier function and promoting immune tolerance over inflammation. On the contrary, diets low in fiber, and high in fat and sugar have been shown to promote a microbiota associated with development of intestinal and extra-intestinal diseases such as food allergy, inflammatory bowel disease and metabolic disease. Despite the established role of the diet and microbiota in regulation of intestinal health, many fundamental questions remain to be answered and the challenges ahead lies in 1) identify the molecular mechanisms involved in mucus impairment, 2) further characterization of the bacteria and bacterial metabolites that influence goblet cell function and mucus properties, 3) establish the importance of different type of goblet cells in the control of mucus production and intestinal immunity, 4) assessment of the role of peripheral organs such as the liver in regulation of the mucus barrier *via* production of bioactive compounds further metabolized by the microbiota, 5) characterization of the mechanisms by which specific dietary components influences intestinal homeostasis, and how diet induced changes in the microbiota influences the ability of the intestine to maintain tolerance to the luminal content, 6) evaluation of the therapeutical potential of dietary fiber/bacterial metabolite supplements in restoration of mucus barrier defects and loss of oral tolerance, and 7) deeper investigation of the phage-mucus interactions.

## Author contributions

FS and JG conceived, supervised, and revised the first draft of the review. FS, EN, DS, and JG wrote and edited the review. All authors read and approved the final version of the manuscript.

## Funding

This work was supported by the WoM Lundgren grant (2022-3916), the Swedish Research Council (2019-01134 and 2020-01588), the Crohn’s and Colitis foundation (580014), and the Swedish Society for Medical Research (PD20-0168).

## Acknowledgments

We thank Malin E. V. Johansson for critically revising the review, and Ana Sofia de Jesus Vaz Luis for providing inputs and literature. **Figures 1** and **2** are created with BioRender.com

## Conflict of interest

The authors declare that the research was conducted in the absence of any commercial or financial relationships that could be construed as a potential conflict of interest.

## Publisher’s note

All claims expressed in this article are solely those of the authors and do not necessarily represent those of their affiliated organizations, or those of the publisher, the editors and the reviewers. Any product that may be evaluated in this article, or claim that may be made by its manufacturer, is not guaranteed or endorsed by the publisher.

## Abbreviations

Aldh1A2, Adehydrogenase 1A2; AhR, Ahryl carbon receptor; AGR, Anterior gradient; AMPs, Anti-microbial peptides; CLCA1, Chloride channel accessory; Convr, Conventionally raised; DCs, Dendritic cells; FCGBP, Fc fragment of IgG binding protein; GPRs, G protein coupled receptors; GI, Gastrointestinal; GF, Germ-free; GCs, Goblet cells; HF, High-fat; HDAC, Histone deacetylase; HMOs, Human milk oligosaccharides; IDO1, Indoleamine 2,3-dioxygenase 1; ILCs, Innate lymphoid cells; IL, Interleukin; IECs, Intestinal epithelial cells; IELs, intra epithelial lymphocytes; LP, Lamina propria; LPS, Lipopolysaccharide; MUC2, Mucin2; NKT, Natural killer T cells; PGN, Peptidoglycan; IgA, Immunoglobulin A; SCFAs, Short-chain fatty acids; Th, T helper cells; Tregs, T regulatory cells; TLRs, Toll-like receptors; UC, Ulcerative colitis; WD, Western diet; ZG16, Zymogen granule protein 16.
